# Identification of colon cancer subtypes based on multi-omics data—construction of methylation markers for immunotherapy

**DOI:** 10.3389/fonc.2024.1335670

**Published:** 2024-01-22

**Authors:** Benjie Xu, Jie Lian, Xiangyi Pang, Yue Gu, Jiahao Zhu, Yan Zhang, Haibo Lu

**Affiliations:** ^1^ Department of Outpatient Chemotherapy, Harbin Medical University Cancer Hospital, Harbin, China; ^2^ School of Life Science and Technology, Computational Biology Research Center, Harbin Institute of Technology, Harbin, China; ^3^ College of Pathology, Qiqihar Medical University, Qiqihar, China

**Keywords:** colon cancer, DNA methylation, microsatellite status, immunotherapy, specific DNA methylation markers

## Abstract

**Background:**

Being the most widely used biomarker for immunotherapy, the microsatellite status has limitations in identifying all patients who benefit in clinical practice. It is essential to identify additional biomarkers to guide immunotherapy. Aberrant DNA methylation is consistently associated with changes in the anti-tumor immune response, which can promote tumor progression. This study aims to explore immunotherapy biomarkers for colon cancers from the perspective of DNA methylation.

**Methods:**

The related data (RNA sequencing data and DNA methylation data) were obtained from The Cancer Genome Atlas (TCGA) and UCSC XENA database. Methylation-driven genes (MDGs) were identified through the Pearson correlation analysis. Unsupervised consensus clustering was conducted using these MDGs to identify distinct clusters of colon cancers. Subsequently, we evaluated the immune status and predicted the efficacy of immunotherapy by tumor immune dysfunction and exclusion (Tide) score. Finally, The Quantitative Differentially Methylated Regions (QDMR) software was used to identify the specific DNA methylation markers within particular clusters.

**Results:**

A total of 282 MDGs were identified by integrating the DNA methylation and RNA-seq data. Consensus clustering using the K-means algorithm revealed that the optimal number of clusters was 4. It was revealed that the composition of the tumor immune microenvironment (TIME) in Cluster 1 was significantly different from others, and it exhibited a higher level of tumor mutation burdens (TMB) and stronger anti-tumor immune activity. Furthermore, we identified three specific hypermethylation genes that defined Cluster 1 (PCDH20, APCDD1, COCH). Receiver operating characteristic (ROC) curves demonstrated that these specific markers could effectively distinguish Cluster 1 from other clusters, with an AUC of 0.947 (95% CI 0.903-0.990). Finally, we selected clinical samples for immunohistochemical validation.

**Conclusion:**

In conclusion, through the analysis of DNA methylation, consensus clustering of colon cancer could effectively identify the cluster that benefit from immunotherapy along with specific methylation biomarkers.

## 1 Introduction

Colon cancer, being one of the prevalent malignancies of the digestive system, exhibits the second highest mortality rates globally and ranks third in terms of incidence. This medical challenge poses a significant threat to human health (1). Systemic therapy is the primary treatment for advanced colon cancers. Unfortunately, the five-year overall survival (OS) is currently estimated at only 30% (2). Immunotherapy significantly prolonged the survival of patients with deficiency of mismatch repair (dMMR) or microsatellite instability – high (MSI -H) (3–5). However, the detection rate of dMMR or MSI-H only accounts for 5%-10% in colon cancers (6, 7). Additionally, approximately 25% of detected patients do not benefit from immunotherapy (8). It is worth noting that some patients with microsatellite stability (MSS) also experienced prolonged OS after immunotherapy (9). In a word, microsatellite status had certain limitations as a criterion for predicting the effectiveness of immunotherapy. The current research priorities are focused on identifying additional biomarkers in order to expand the accessibility of immunotherapy.

DNA methylation is a crucial epigenetic modification that plays a substantial function in regulating gene expression (10, 11). DNA methylation is the process of adding a methyl group to the 5’ positions of a cytosine and guanosine (CpG) with the participation of DNA methyltransferase (DNMT). CpGs are typically abundant in the promoter region of CpG islands. Hypermethylation of promoter region always leads to the silencing of tumor suppressor gene expression (12, 13) and DNA methylation plays a regulatory role in tumor antigen presentation and the release of immune factors (14–16). To summarize, aberrant DNA methylation, especially hypermethylation of promoter regions, is frequently associated with altered anti-tumor immune responses, leading to tumor progression.

Currently, diagnostic and prognostic related methylation markers have been identified in colon cancer (17). This study is the first to identify immunotherapy biomarkers for colon cancer from the perspective of methylation. Through the identification of methylation-driven genes (MDGs), performing cluster analysis and verified by clinical samples. We identify a specific cluster of colon cancer that could be benefit from immunotherapy. Furthermore, we discovered beneficial-cluster of specific DNA methylation markers that can be used as a valuable supplement to the microsatellite status.

## 2 Materials and methods

### 2.1 Data acquisition and processing

RNA sequencing (RNA-Seq) data, somatic mutation data, clinicopathological data (including microsatellite status) of colon cancers were downloaded from The Cancer Genome Atlas (TCGA) database (https://portal.gdc.cancer.gov). DNA methylation data (Illumina Human Methylation 450) were obtained from the UCSC XENA database (https://xena.ucsc.edu/). For each probe site, methylation levels ranged from 0 (fully unmethylated) to 1 (fully methylated). Firstly, the DNA methylation data was screened, eliminating probes that exhibited missing information in over 70 percent of the samples. Secondary, probes in the sex chromosome and single nucleotide polymorphisms were also excluded. Finally, the K-nearest neighbors (KNN) algorithm was utilized to impute the missing values, implemented through the (knn) imputation procedure. Since DNA methylation in the promoter region could regulate expression of genes, we specifically analyzed the CpGs in the promoter region. The promoter was defined as the upstream 2.5 kb to downstream 0.5kb region of the transcription start site. For the expression data, we focused on analyzing the protein-encoding mRNA.

### 2.2 Differential analysis and identification of DNA methylation-driven genes

Between tumor and normal tissues, RNA-Seq data was analyzed using the “Deseq2” package implemented in R to detect differentially expressed genes (DEGs). The criteria for DEGs were set at a threshold of *P<* 0.05 and | log2FC | > 1 (18). On the other hand, methylation data was analyzed using the limma package to identify differentially methylated genes (DMGs), with a set of *P*< 0.05 (19). The overlapped portion of the DEGs and DMGs, representing differentially expressed genes with aberrantly methylation, which were visualized using a Venn diagram.

The DNA methylation and RNA-Seq data of differentially expressed genes with aberrantly methylation were integrated for correlation analysis using the Pearson coefficient. A threshold of Pearson coefficient< -0.3 and *P*< 0.05 was set to identify MDGs for further analysis. The scatter plot of MDGs was created using ggplot2 in R (20).

### 2.3 Analysis of function enrichment construction of the PPI network

The”clusterProfiler”, “org.Hs.eg.db”, and “enrichplot” R package were used to evaluate the most significantly enriched function and pathway. The Gene Ontology (GO) and Kyoto Encyclopedia of Genes and Genomes (KEGG) analysis considered results with *P<* 0.05 and *q*< 1 as the differentially enriched (21, 22). These results were visualized using the ‘ggplot2’ R package.

To construct the Protein-Protein interaction network (PPI), the MDGs were uploaded to the Interactive Gene/protein Retrieval Tool Database (STRING) (https://string-db.org/). The identification of key genes and major modules in the PPI network was performed using the Cytoscape software.

### 2.4 Consensus clustering analysis

Consensus clustering was performed (ConsensusClusterPlus R package) to identify clusters of colon cancer with distinct molecular features (23). The K-means algorithm and Euclidean distance were employed in clustering.


$$d=\sqrt{\sum_{k=1}^{N}{\left(x_{k}-y_{k}\right)}^{2}}$$

The optimal number of clusters (k) were tested from 2 to 9 in this study. The procedure of clustering was conducted over 1000 iterations, in which 80% of the data was sampled in each iteration. The selection criteria for determining the optimal k value included the cluster’s internal consistency, low coefficient of variation, and stability of the area under the cumulative distribution function (CDF) curves. The optimal number of clusters was determined using Principal component analysis (PCA) in this study. The Cumulative Density Function (ECDF) was used to calculate the area between 0.1 and 0.9 of the X-axis, the k value corresponding to the minimum ECDF area was the optimal number of clusters. Subsequently, survival analysis was used to evaluate the prognosis. The statistical significance among the clusters was evaluated using the log-rank test, with *P*< 0.05 considered significant. The performance of classification was evaluated using the receiver operating characteristic (ROC) curves.

### 2.5 Evaluation of the immune status among different colon cancer clusters

Unsupervised consensus clustering was performed to identify distinct clusters of colon cancers. Subsequently, we evaluated the immune status of these clusters.

The stromal score and immune score were calculated using the ESTIMATE algorithm based on expression data, which represented the presence of stromal and immune cells. The sum of stromal and immune scores was used as the estimate score to evaluate tumor purity. This evaluation was performed using the R language ‘estimate’ package (24). Immune checkpoint inhibitors (ICIs) could guide the immunotherapy of colon cancers. This study statistically analyzed the expression of the most common ICIs (PD-1, PD-L1, PD-L2, CTLA4, LAG3) among different clusters. Additionally, we quantified the abundance of tumor-infiltrating immune cells (TIICs) using the CIBERSORT algorithm (25). This study analyzed the immune status of clusters to determine if there was statistical difference, with *P*< 0.05 considered significant.

The Cancer-Immunity Cycle, commonly referred to as the anti-cancer immune response, consists of seven steps. These steps begin with the release of cancer cell antigens and end with the killing of cancer cells (26). The website Tracking Tumor Immunophenotype (TIP) (http://biocc.hrbmu.edu.cn/TIP) specialized in the study of the Cancer-Immunity Cycle and had calculated the immune activity scores for each step through large sample analysis (27). In the present study, we collected immune activity scores of colon cancer samples from the TIP website to analyze the differences in clusters within the Cancer-Immunity Cycle.

In addition, the somatic mutation data of colon cancers were analyzed and visualized using the R language “maftools” package (28). We specifically accessed the mutation frequencies and the level of tumor mutation burdens (TMB) from different clusters. The statistical results were depicted through the boxplots, with *P*< 0.05 considered significant.

### 2.6 Prediction of immunotherapy by Tide score

Immune evasion, a key factor in tumor development, significantly contributed to the failure of immunotherapy. There are two mainly mechanisms in the process of immune evasion. Firstly, tumors characterized by a substantial infiltration of cytotoxic T lymphocytes (CTLs) exhibited the induction of T cell inactivation, leading to dysfunction of immune cells. Secondly, in tumors with diminished levels of CTLs, T cell infiltration was prevented and the ability of killing tumor cells was weakened (29, 30). Based on sequencing data, the Tumor Immune Dysfunction and Exclusion (TIDE) algorithm (http://tide.dfci.harvard.edu/) could reveal the characteristics of tumor immune evasion. By utilizing CTLs observed in tumor samples, the TIDE score can be calculated to predict the efficacy of immunotherapy. Specifically, In the case of melanoma, the TIDE score demonstrates greater predictive accuracy compared to biomarkers like PD-L1 (31). Consequently, the present study employed the TIDE score to predict the efficacy of immunotherapy within distinct clusters.

### 2.7 Identification of specific DNA methylation markers

In our study, we utilized Quantitative Differentially Methylated Regions (QDMR) software to identify the specific DNA methylation CpGs within particular clusters of colon cancer. QDMR was an effective tool developed based on the Shannon entropy model, which allowed for the detection of DMRs across multiple DNA methylation profiles (32). The entropy difference reflected the influence of sample S on the overall methylation difference:


$$\Delta H_{r/s}=H_{Q/}\bar{S}-H_{Q}$$

When region r is specifically methylated in sample S, the value of $△H_{r/S}$ is greater than 0. The categorical sample-specificity $CS_{r/S}$ can be defined as:


$$CS_{r/S}=\left\{\begin{array}{l} \Delta H_{r/S}\times {\text{sign}}_{r,S},\Delta H_{r,S}>0\\ 0,\Delta H_{r/S}\leq 0 \end{array}\right\}$$

Therefore, $CS_{r/S}$ can be utilized to analyze identify specific DNA methylation markers in samples. $CS_{r/S}$ greater than 0 indicates specifically hypermethylated, while a value less than 0 indicates specifically hypomethylated.

### 2.8 Immunohistochemistry

To investigate the prediction of immunotherapy response using specific markers, our study conducted a review of colon cancer patients in our center. We retrospectively collected their follow-up and treatment records, including postoperative recurrence, immunotherapy duration and cycles, and efficacy evaluation. These records successfully helped us to screen out the validation objects. The corresponding tumor paraffin sections were analyzed by immunochemistry. After roasting the sections at 60°C for 20 minutes, they were deparaffinized with xylene and rehydrated. The antigen was then recovered from the sections by heating the EDTA buffer (100°C for 15 minutes) and the endogenous peroxidase activity was inactivated using 3% H_2_O_2_ (10 minutes). The sections were treated with 5% BSA and incubated at room temperature for 1 hour. They were then incubated overnight at 4°C with primary antibodies (APCDD1, 1:20, Thermo, PA535063; PCDH20, 1:25, Thermo, PA598605). After washing the sections with PBS, secondary antibodies (1:500) were added to sections and incubated at room temperature for 1 hour. Finally, color development was achieved using the DAB kit (CWBIO-CW0125), and hematoxylin solution was used for counterstaining the paraffin sections. An open-source biological image analysis platform (Fiji/ImageJ) was utilized for analyzing the sections. The evaluation of immunohistochemical was based on both the staining intensity and the percentage scores. Staining intensity was graded on a scale of 0 (absent), 1 (weak), 2 (moderate), and 3 (marked), while the percentage scores was determined by the proportion of stained cells in a chosen field: 1 (0-25%), 2 (26-50%), 3 (51-75%), and 4 (76-100%). Each tumor sample was independently scored by two observers, and the results were reported as the mean score (ranging from 0 to 14).

### 2.9 Statistical analysis

The statistical analyses in this study were performed by R software (4.13 version) and GraphPad Prism 8 (GraphPad Software, La Jolla, CA, USA). The correlation between the two variables was assessed by the Pearson coefficient. For continuous data, the independent Student’s t-test was conducted. Additionally, the chi-square test was applied to analyze categorical data. To compare non-normally distributed variables across clusters, we utilized the Wilcoxon test. The Kruskal–Wallis test was used for multiple groups. Statistical significance was determined based on a two-tailed P-value of less than 0.05 and we also reported the hazard ratios (HRs) and 95% confidence intervals (CIs).

## 3 Results

### 3.1 DNA methylation-driven genes

**Figure 1:**
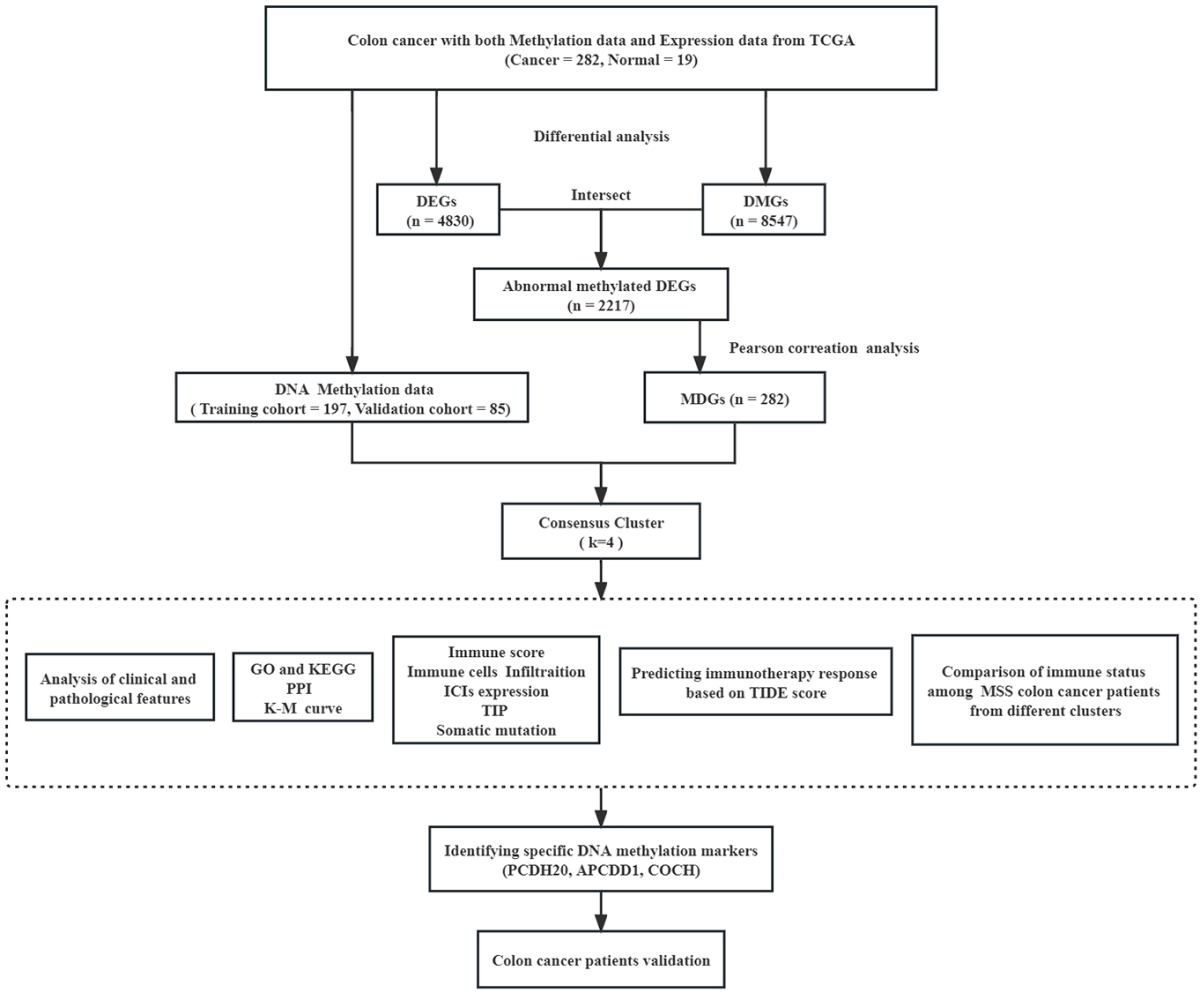
Flow diagram and analytic procedure of our study.

**Figure 2:**
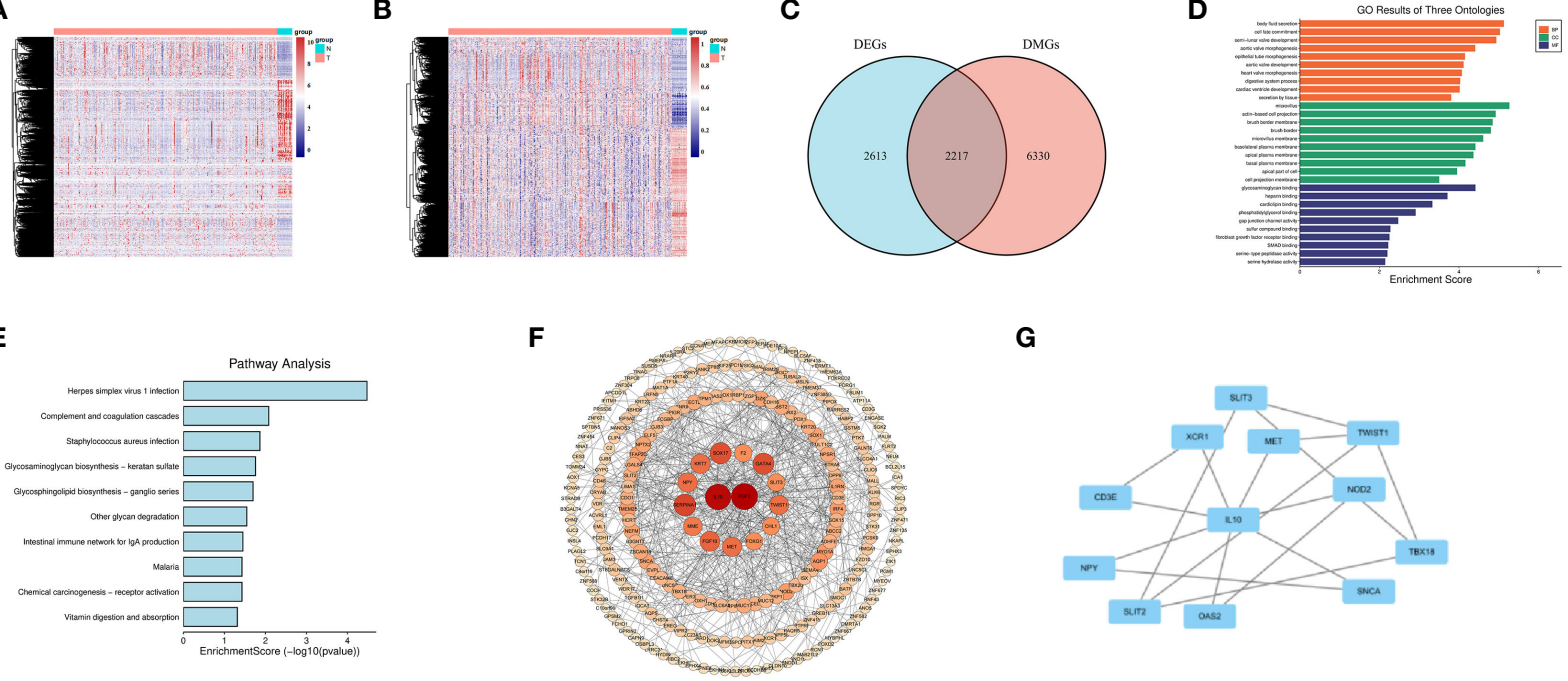
Screening for DNA Methylation-Driven Genes (MDGs). **(A)** Heatmap of Differentially Expressed Genes (DEGs) in normal samples and colon cancer samples. **(B)** Heatmap of Differentially Methylated Genes (DMGs) in normal samples and colon cancer samples. **(C)** Venn diagram for overlapping of DEGs and DMGs. **(D)** Gene Ontology (GO) enrichment results of three ontologies (including biological processes, cellular components, and molecular functions) of MDGs. **(E)** Kyoto Encyclopedia of Genes and Genomes (KEGG) enrichment analysis of MDGs. **(F, G)** Protein-Protein Interaction (PPI) network of MDGs.

The flow diagram and analytic procedure are shown in **Figure 1**. The data of colon cancers were downloaded from the relevant database. A total of 301 samples had both DNA methylation and RNA-seq data (282 tumor and 19 normal). For RNA-seq data, we selected mRNA (19,938 genes) for differential analysis, detecting a total of 4830 DEGs at last. The expression of DEGs between colon cancers and normal samples was showed in the heatmap (**Figure 2A**). In the case of DNA methylation data, we selected CpGs (164,610 sites) and corresponding genes (18,510 genes) in the promoter region for difference analysis. If multiple CpGs correspond to the same gene, the mean value of β was selected to represent the methylation level of that gene. Similarly, a total of 8547 DMGs were detected. The heatmap showed the methylation of DMGs between colon cancers and normal samples (**Figure 2B**). Subsequently, 2217 differentially expressed genes with aberrantly methylation were identified by overlapping DEGs and DMGs (**Figure 2C**, **Table S1**).

We conducted correlation analysis by integrating the DNA methylation and RNA-seq data of 2217 differentially expressed genes with aberrantly methylation in colon cancers. The Pearson coefficient was utilized to access the correlation. Finally, we identified 282 MDGs for further analysis based on a Pearson coefficient< -0.3 and *P<* 0.05 (**Table S2**).

### 3.2 Function enrichment and PPI network construction

We conducted GO and KEGG analyses on 282 MDGs to analyze their potential functions and pathways. The results of GO analysis were significantly enriched in fibroblast growth factor receptor binding, digestive system process, etc(*P*<0.05, **Figure 2D**). Additionally, KEGG pathways analysis revealed significant enrichment in virus infection (Herpes simplex virus 1, Staphylococcus aureus), intestinal immune network for IgA production, etc(*P*<0.05, **Figure 2E**).

A PPI network was conducted to illustrate the interactions and connections of 282 MDGs in colon cancers. The degree algorithm was employed to determine the significance of different genes in the PPI network, while the size and color of nodes were utilized for visualization. Among these genes, IL-10 and FGF2, as core genes, playing a crucial role in the interconnection network (**Figure 2F**). By employing the MCODE plugin in Cytoscape, we identified key sub-networks within the PPI network, which included genes such as IL-10, CD3E, MET, and others that were associated with anti-tumor immune response (**Figure 2G**).

Overall, our findings indicated that strong interconnections among the 282 MDGs in colon cancers, with IL-10 and FGF2 acting as core genes that are closely linked to tumor angiogenesis and anti-tumor immune response.

### 3.3 Consensus clustering in colon cancers

In this study, we performed consensus clustering based on the β values of the 282 MDGs to identify distinct DNA methylation molecular clusters of colon cancers. Subsequently, 282 samples were randomly divided into training (n = 197) and validation cohorts (n = 85) in a 7:3 proportion. The Chi-square test indicated that the clinicopathologic features of the training and validation cohorts were evenly distributed (**Table S3**).

**Figure 3:**
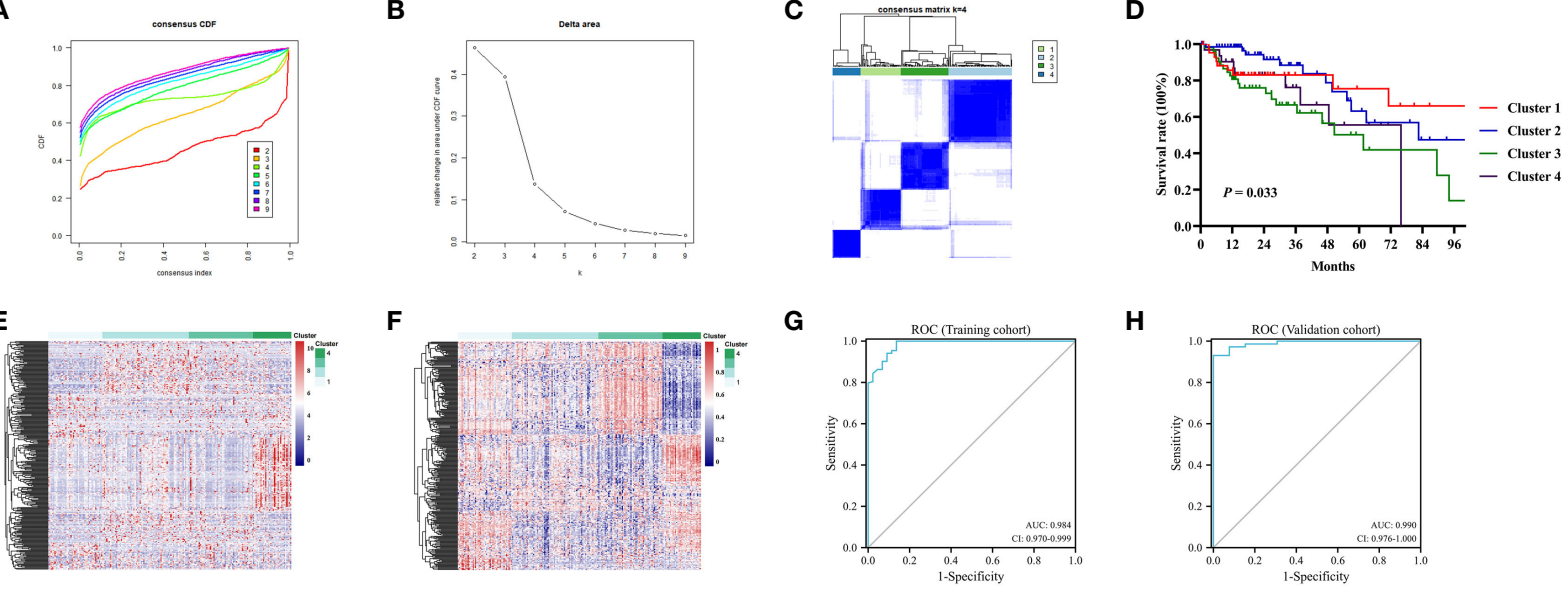
Consensus analysis for DNA methylation based on 282MDGs. **(A)** Consensus cumulative distribution function (CDF) of different clusters for colon cancer. **(B)** Delta area curve of consensus clustering. **(C)** Consensus clustering matrix for colon cancer at k = 4. **(D)** The survival curves in four DNA methylation clusters. **(E)** Heatmap of gene expression levels of 282 MDGs in the four clusters. **(F)** Heatmap of methylation levels of 282 MDGs in the four clusters. **(G)** Receiver operating characteristic (ROC)curves of the 282 MDGs in distinguishing four clusters in the train cohort. **(H)** ROC curves in the validation cohort.

The K-means algorithm was utilized for consensus clustering. According to the relative alteration observed under the CDF curve, the PCA method was finally employed to ascertain the optimal number (**Figures 3A, B**, **Figure S1A, B**). It was found that K = 4 was the optimal clustering with best stability (**Figure 3C**, **Figure S1C**). Which were termed Cluster 1 (44 patients, 22.4%), Cluster 2 (70 patients, 35.5%), Cluster 3 (52 patients, 26.4%) and Cluster 4 (31 patients, 15.7%), respectively. The K-M survival analysis revealed significant difference among the four clusters (*P*<0.05, **Figure 3D**, **Figure S1D**). The heatmaps displayed the significant differences among the clusters in terms of gene expression and methylation levels for 282 MDGs (**Figures 3E, F**). Moreover, both the training cohort and the validation cohort exhibited excellent performance in discriminating the clusters of colon cancer using the MDGs, with an AUC of 0.984 (95%CI 0.970-0.999) and 0.990 (95%CI 0.976-1.000), respectively (**Figures 3G, H**). The chi-square test revealed significant differences in clinicopathological characteristics among these clusters. Patients in Cluster 1 were found to be associated with age (*P*<0.001) and microsatellite status (*P*<0.001). The remaining clinicopathological characteristics showed no distribution differences among clusters (**Table S4**). Similar distribution was found in the validation cohort (**Table S5**).

### 3.4 Distinct immune status among colon cancer clusters

**Figure 4:**
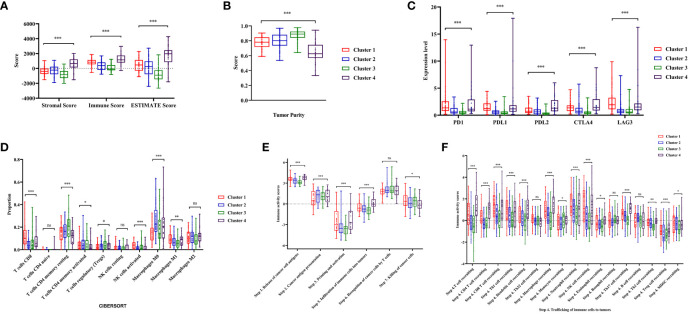
Analysis of tumor immune microenvironment and immune status during four clusters. **(A, B)** Comparisons of stromal score, immune score, ESTIMATE score and tumor purity during four clusters. **(C)**The expression level of immune checkpoints (PD1, PDL1, PDL2, CTLA4, LAG3) in the four clusters of colon cancer. **(D)** The abundance of immune cells in the four clusters of colon cancer patients evaluated by CIBERSORT algorithm. **(E, F)** Comparisons of the immune activity score from Tracking Tumor Immunophenotype (TIP) database in four clusters. ‘ns’ means *P* > 0.05, * means *P*< 0.05, ** means *P*< 0.01, *** means *P*< 0.001.

Through a series of analyses, significant differences in the immune status of different clusters were revealed. First, the composition of tumor immune microenvironment (TIME) was analyzed. Significant differences were observed in the immune score, stromal score, and tumor purity among clusters of colon cancer (*P*<0.001, **Figures 4A, B**, **Figure S1E, F**). Cluster 1 exhibited with a relative higher immune score and lower tumor purity, indicating a greater infiltration of immune cells. Subsequently, the expression levels of several common ICIs (PD1, PDL1, PDL2, CTLA4, LAG3) were compared among clusters. The findings illustrated that the ICIs expression in cluster 1 exhibited a significantly greater level compared to cluster 2 and 3 (*P*<0.001, **Figure 4C**, **Figure S1G**). Finally, the CIBERSORT algorithm was employed to visualize the infiltration abundances of TIICs in colon cancers. In cluster 1, there was a significant abundance of CD8+T cells, activated natural killer cells and M1 macrophages, which were associated with anti-tumor immune response, compared to other clusters. The abundant of immunosuppression-related Tregs cells in cluster 1 was relative lower (*P*<0.05, **Figure 4D**, **Figure S2A**).

The immune activity scores of colon cancers were used to evaluate the Cancer-Immunity Cycle. The results showed significant differences in the procedure of Cancer-Immunity Cycle among the clusters. The mean scores of Step1 (release of specific cancer cell antigens), Step 3 (priming and activation) and Step 7 (killing cancer cells) were significantly higher in Cluster 1, compared to Cluster 2 and 3 (*P*<0.01, **Figure 4E**). Additionally, Step 4 (trafficking of immune cells to tumors), which played a major role in the Cancer-Immunity Cycle, showed a higher abundance of CD8+ T cells, macrophages, and natural killer cells in Cluster 1 (*P*<0.05, **Figure 4F**).

These results indicated that the composition of the TIME in Cluster 1 was significantly different from others, and it exhibited a higher level of immune infiltration and stronger anti-tumor immune activity.

### 3.5 Somatic mutation landscape of clusters

**Figure 5:**
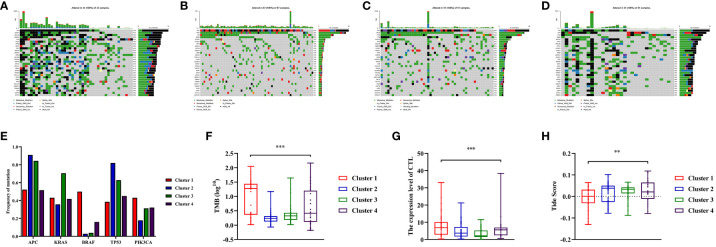
Somatic variations features during clusters of colon cancer and predicting the response to immune therapy based on Tumor Immune Dysfunction and Exclusion (TIDE) score. **(A–D)** Waterfall plots showed somatic mutation landscape and the top 10 mutated in four clusters. **(E)** Comparisons of mutation status of APC, KRAS, BRAF, TP53 and PIK3CA during different clusters of colon cancer patients. **(F)** Comparisons of Tumor Mutation Burdens (TMB) level of the four clusters. **(G)** Comparisons of cytotoxic T lymphocytes (CTL) level of the four clusters. **(H)** Comparisons of Tide score for predicting the likelihood of response to immune therapy of different clusters. ** means *P*< 0.01, *** means *P*< 0.001.

In previous research, the critical involvement of genetic mutations in the initiation and progression of colon cancers has been investigated. Consequently, we conducted an analysis of somatic mutation information to investigate the genomic variations within distinct clusters. Among these clusters, APC, TP53, and PIK3CA were the most common gene aberrations (**Figures 5A-D**). This study specifically focused on the distribution of the mutation frequency of KRAS and BRAF genes across different clusters, which were important for targeted therapy in patients with colon cancer. Interestingly, the prevalence of BRAF mutations was significantly higher in Cluster 1 compared to other clusters, and a similar trend was observed for KRAS mutations in Cluster 3 (*P*<0.05, **Figure 5E**, **Figure S2B**). In addition, we also assessed the TMB, a predictive biomarker for immunotherapy. It was found that patients in Cluster 1 had a significantly higher level of TMB compared to other clusters (*P*<0.001, **Figure 5F**, **Figure S2C**). This suggested that Cluster 1 may exhibit a better response to immunotherapy (33).

### 3.6 Prediction of immunotherapy response among colon cancer clusters

The TIDE score was calculated to predict the efficiency of immunotherapy by analyzing the correlation between gene expression and CTLs infiltration level. In this study, the average expressions of CD8A, CD8B, GZMA, GZMB, and PRF1 genes were used to represent level of CTL in colon cancers. Based on the findings, it was observed that the Cluster 1 exhibited noticeably higher level of CTLs compared to Cluster 2 and 3 (*P*<0.001, **Figure 5G**, **Figure S2E**). Furthermore, a higher Tide score indicated a greater likelihood of immune evasion and no benefit from immunotherapy. It was found that Cluster 1 was more likely to benefit from immunotherapy as its score was significantly lower compared to Cluster 2 and 3 (*P*<0.05, **Figure 5H**, **Figure S2F**).

In this study, we compared the immune status of different clusters and found no statistical difference between Cluster 1 and Cluster 4 in the composition of TIME and the expression of ICIs. However, there were significant differences in the TMB level between Cluster 1 and Cluster 4. In addition, we used the TIDE score to predict immunotherapy responses in different clusters and found that Cluster 1 had a significantly lower score compared to Cluster 2 and 3. While, Cluster 4 exhibited a TIDE score that did not exhibit significant difference from that in Cluster 2 and 3 (*P*>0.05).

### 3.7 Comparison of immune status among different clusters of MSS patients

**Figure 6:**
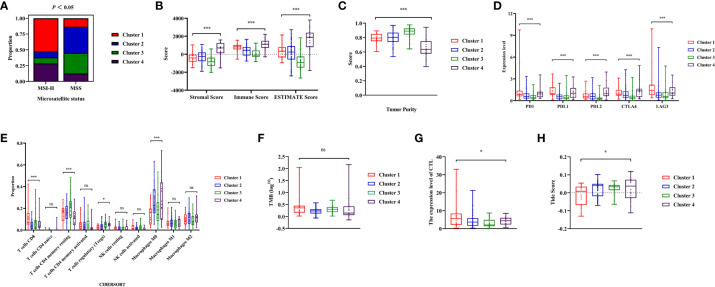
Analysis of immune status during microsatellite stability (MSS) patients from the four clusters. **(A)** The distribution frequency of microsatellite status during four clusters. **(B, C)** Comparisons of stromal score, immune score, ESTIMATE score and tumor purity during MSS patients from different clusters. **(D)** The expression level of immune checkpoints (PD1, PDL1, PDL2, CTLA4, LAG3) during MSS patients from different clusters. **(E)** Comparisons of the abundances of immune cells evaluated by CIBERSORT algorithm during MSS patients from different clusters. **(F)** Comparisons of Tumor Mutation Burdens (TMB) level of the four clusters. **(G)** Comparisons of cytotoxic T lymphocytes (CTL) level of the four clusters. **(H)** Comparisons of Tumor Immune Dysfunction and Exclusion (TIDE) score for predicting the response of immune therapy during MSS patients. ‘ns’ means *P*> 0.05, * means *P*< 0.05, *** means P< 0.001.

The study has confirmed that Cluster 1 was more likely to benefit from immunotherapy. It was observed that the distribution frequency of dMMR/MSI-H in Cluster 1 was significantly higher (50%) compared to other clusters (*P*<0.05, **Figure 6A**, **Figure S2D**). To investigate whether the distinct distribution of dMMR/MSI-H contributed to the varying immune status of each cluster, data from MSS patients from clusters were collected for further analysis.

It was found that the stromal, immune score and tumor purity were significantly different among clusters (P< 0.01, **Figures 6B, C**). Cluster 1 exhibited higher immune score with increased immune cell infiltration, while having relatively lower tumor purity. The expression level of ICIs in cluster 1 were significantly higher than cluster 2 and 3(*P*<0.05, **Figure 6D**). Among the clusters, Cluster 1 showed significantly higher abundance of CD8+T cells compared to others (*P*<0.001, **Figure 6E**). Moreover, the abundance of immunosuppression-related Tregs cells among clusters varied statistically among clusters, with Cluster 1 showing relatively lower infiltration (*P*<0.05, **Figure 6E**). Although there was no significant difference in the TMB levels among clusters of colon cancer (*P*=0.051, **Figure 6F**), the average of TMB in Cluster 1 was higher than in other clusters. Finally, the Tide score was calculated using CTL levels to predict immunotherapy response. The CTL levels of each cluster showed significant differences (*P*<0.05, **Figure 6G**), with cluster 1 having the highest CTL level. According to the Tide score, it was anticipated that Cluster 1 patients had a higher probability of experiencing favorable outcomes with immunotherapy, even among those with MSS status (*P*<0.05, **Figure 6H**).

In summary, Significant differences in the TIME of MSS patients were observed from different clusters. MSS patients in Cluster 1 exhibited a better immune status, making them more suitable for immunotherapy.

### 3.8 Identification specific DNA methylation markers

**Figure 7:**
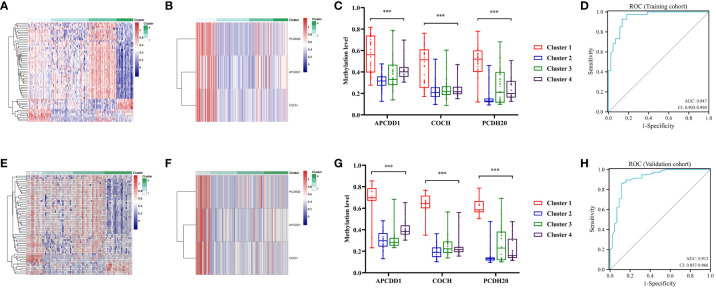
Specific methylation sites for each DNA methylation cluster. **(A)** The heatmap for the specific sites during four DNA methylation clusters in the training cohort. **(B)** The heatmap for the three specific sites (PCDH20, APCDD1, COCH) of cluster 1 during four clusters in the training cohort. **(C)** Comparisons of the methylation level for the three specific sites (PCDH20, APCDD1, COCH) during four clusters in the training cohort. **(D)** Receiver operating characteristic (ROC) curves of the three specific sites (PCDH20, APCDD1, COCH) in distinguishing the Cluster1 from other clusters in the train cohort. **(E)** The heatmap for the specific sites during four clusters in the validation cohort. **(F)** The heatmap for the three specific sites of Cluster 1 during four clusters in the validation cohort. **(G)** Comparisons of the methylation level for the three specific sites during four clusters in the validation cohort. **(H)** ROC curves of the three specific sites in distinguishing the Cluster 1 from other clusters in the validation cohort. *** means *P*< 0.001.

The QDMR software was used to identify the specific methylation genes that characterized distinct DNA methylation clusters of colon cancers. The average DNA methylation level of samples for all 282 MDGs was calculated, resulting in a 282*9dimensional matrix which was then input into QDMR. A standard deviation (SD) parameter of 0.3 was set to identify the specific markers for each cluster. Ultimately, 56 specific methylation genes were identified (**Table S6**). A heatmap was generated based on these specific methylation genes, clearly illustrating the differentiation among clusters (**Figures 7A, E**). Each cluster had its own unique set of specific methylation genes. Of particular interest were the three specific hypermethylation genes that defined Cluster 1 (PCDH20, APCDD1, COCH). Pearson correlation analysis indicated that methylation in the promoter region regulated the gene expression level of specific markers. The correlation coefficients for PCDH20, APCDD1, and COCH were -0.335 (*P*<0.001, **Figure S3A**), -0.309 (*P*<0.001, **Figure S3B**), and -0.329 (*P*<0.001, **Figure S3C**), respectively. The Cluster 1 could be clearly distinguished from the other clusters by three specific makers (**Figures 7B, F**). The boxplot analysis revealed significant differences in methylation levels between Cluster 1 and the remaining clusters (*P*<0.001, **Figures 7C, G**). Finally, ROC analysis showed an AUC of 0.947 (95% CI 0.903-0.990)for distinguishing Cluster 1 in the training cohort (**Figure 7D**) and the specific markers also had an excellent performance in the validation cohort, with an AUC of 0.912 (95% CI 0.8557-0.966) (**Figure 7H**). These findings indicated that the specific methylation genes (PCDH20, APCDD1, COCH) could effectively distinguish Cluster 1 from other clusters. Additionally, we aimed to investigate the relationship between these specific markers and prognosis. Patients were categorized into two groups based on the average expression and methylation levels. Survival analysis revealed that high expression of APCDD1 was associated with a better prognosis (*P*<0.05, **Figure S4A**), while the expression levels of other genes did not show statistical significance in relation to prognosis (**Figure S4B, C**). Interestingly, high methylation levels of APCDD1 were associated with a worse prognosis (*P*<0.05, **Figure S4D**), whereas the methylation levels of the remaining genes did not exhibit any association with prognosis (**Figure S4E, F**). Further subgroup analysis revealed a significant increase in the methylation level of APCDD1 in advanced-stage patients (*P*<0.05, **Figure S5A**). In contrast, the gene expression level exhibited an opposite trend (*P*<0.01, **Figure S5B**).

### 3.9 Immunohistochemical validation

**Figure 8:**
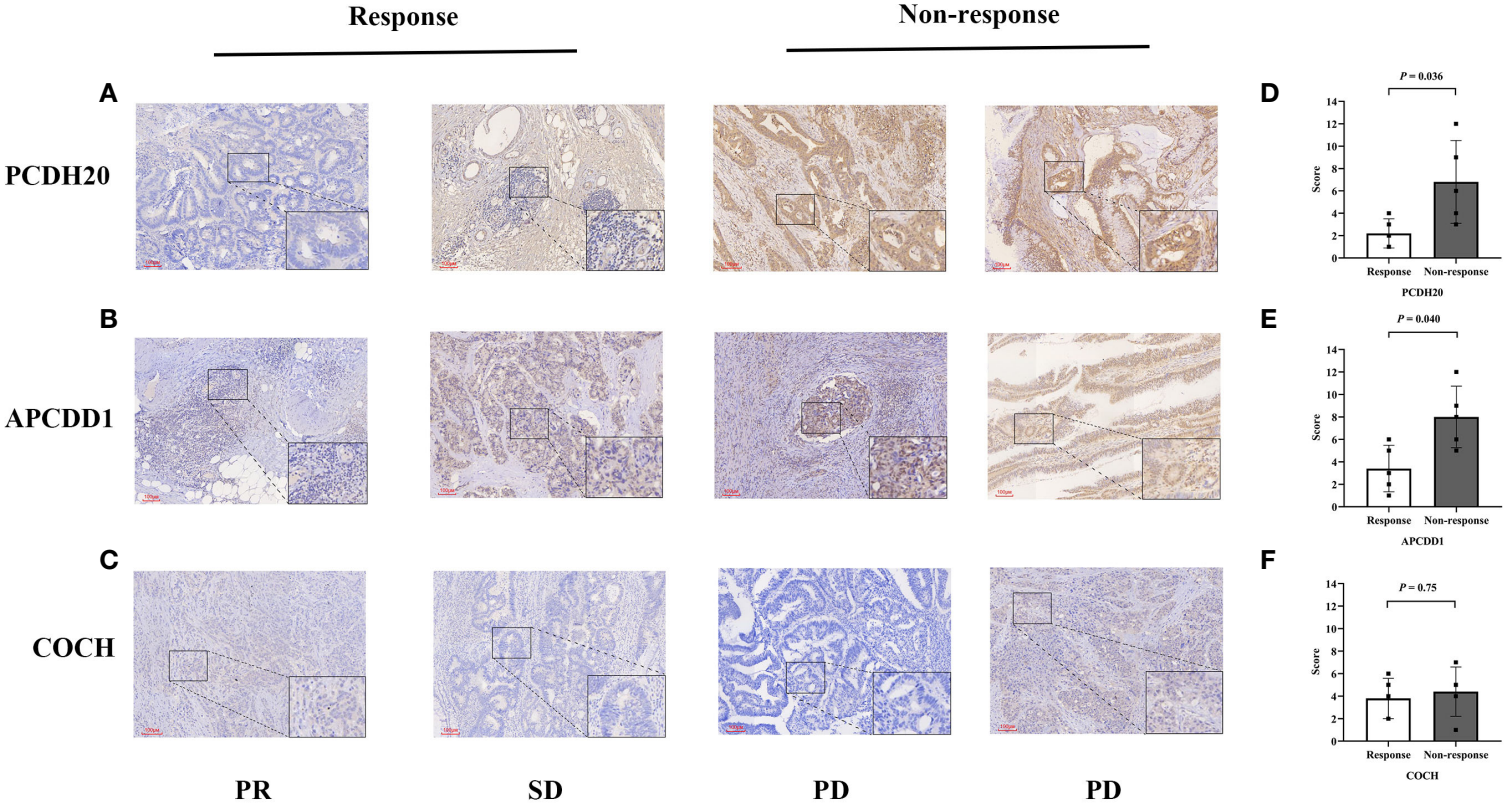
Immunohistochemical validation of colon cancers. **(A-C)** The expressions of (PCDH20, APCDD1 and COCH) in response group and non-response group. **(D, E)** Compared to non-response group, the expressions of (PCDH20 and APCDD1) were down-regulated in response group. **(F)** No significant difference of COCH expression between response group and non-response group.

After screening, we selected the postoperative tumor paraffin sections of 10 patients for immunohistochemical validation. Based on the efficacy evaluation results, the patients were divided into two groups: the response group (partial response (PR), n=2, stable disease (SD), n =3) and the non-response group (progressive disease (PD), n=5) (**Table S7**). The results revealed that the expression scores of biomarkers (PCDH20, APCDD1) were significantly downregulated in the response group compared to the non-response group (*P*<0.05, **Figures 8A, B, D, E**). The hypermethylation of the promoter region could be responsible for the decrease in gene expression levels. In the beneficial-cluster, the methylation levels of PCDH20 and APCDD1 were considerably increased in the benefit cluster, resulting in the repression of the corresponding genes. However, the results indicated that there was no significant difference in the expression of COCH between response group and non-response group (*P* = 0.75, **Figures 8C, F**).

### 3.10 The potential association linking APCDD1 and immune status

**Figure 9:**
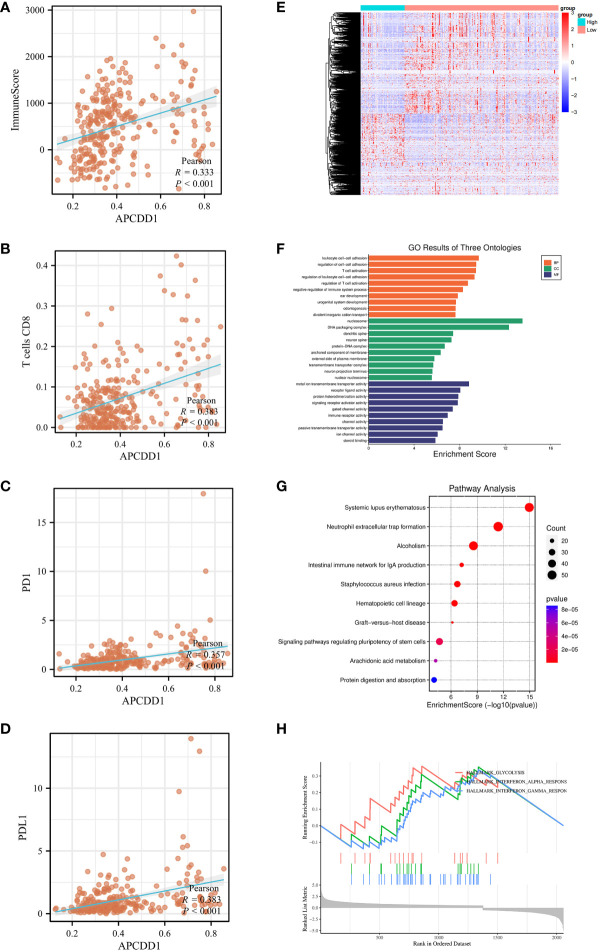
The association of APCDD1 and immune status. **(A)** The methylation level of APCDD1 and immune scores. **(B)** The methylation level of APCDD1 and CD8 T+ cells. **(C)** The methylation level of APCDD1 and PD-1 expressions. **(D)** The methylation level of APCDD1 and PD-L1 expressions. **(E)** Heatmap of Differentially Methylated Genes (DMGs) in different methylation level of APCDD1. **(F)** Gene Ontology (GO) enrichment results of three ontologies (including biological processes, cellular components, and molecular functions) of DMGs. **(G)** Kyoto Encyclopedia of Genes and Genomes **(KEGG)** enrichment analysis of DMGs. **(H)** Gene Set Enrichment Analysis (GSEA) enrichment analysis was carried out on the DMGs between high and low methylation of APCDD1.

To elucidate the underlying mechanisms between markers and immune status. we utilized bioinformatics data to investigate the association between markers and immune scores, immune cell infiltration (CD8+T cells), and immune checkpoints expression (PD-1, PD-L1). Pearson correlation analysis revealed that the methylation level of APCDD1 was positively correlated with immune score (*P*<0.001, 0.333) (**Figures 9A-D**), CD8+T cells infiltration (*P*<0.001, 0.383), PD-1 (*P*<0.001, 0.357), and PD-L1 (*P*<0.001, 0.383). Similarly, the methylation level of COCH exhibited a significant positive correlation with immune scores (*P*<0.001, 0.233), CD8+ T cells infiltration (*P*<0.001, 0.203), PD-1 (*P*<0.01, 0.183), and PD-L1 (*P*<0.001, 0.280) (**Figures S6A-D**). Additionally, the methylation level of PCDH20 showed a significant positive correlation with immune scores (*P*<0.01, 0.192), CD8+ T cells infiltration (*P*<0.001, 0.228), PD-1 (*P*<0.05, 0.129), and PD-L1 (*P*<0.01, 0.185) (**Figures S6G, H**). These findings indicated a significant correlation between the methylation of markers and the immune microenvironment and we chose to focus our research on APCDD1, which demonstrated the strongest correlation with the immune status.

Subsequently, APCDD1 was divided into high and low groups based on methylation level for differential analysis with a set of *P*< 0.05 (**Figure 9E**). The 2058 of DMGs were then subjected to enrichment and analysis. The results of GO analysis were significantly enriched in T cell activation, immune receptor activity (*P*<0.05, **Figure 9F**). Additionally, KEGG pathways analysis revealed significant enrichment in intestinal immune network for IgA production, (*P*<0.05, **Figure 9G**). After conducting a comprehensive Gene Set Enrichment Analysis (GSEA) (h.all.v2022.1.Hs.symbols.gmt), we observed a significant positive association between the differentially expressed genes and the Interferon-GAMMA-Response pathway(*P*<0.05, **Figure 9H**).

## 4 Discussion

The emergence of immunotherapy has marked the beginning of a new era in cancer therapy. However, the current situation presents a challenge with the low detection rate of microsatellite status, which is the primary standard used to guide immunotherapy. Not all patients with dMMR or MSI-H had a response to immunotherapy, a subset of MSS patients could also benefit from immunotherapy. It is essential to search for additional immunotherapy biomarkers as a supplement.

Abnormal DNA methylation modifications are closely associated with the tumor immune microenvironment. This study aimed to identify immunotherapy biomarkers for patients with colon cancer from the perspective of DNA methylation. Firstly, the most crucial step in this research is to utilize DEGs for the identification of MDGs. Our concern lies in the fact that the expression of DMGs is regulated by methylation and remains in a low expression state. Upon applying more stringent thresholds, we observed a substantial reduction in the number of DEGs. However, this reduction came at the cost of decreased sensitivity. The stricter thresholds led to the exclusion of potentially relevant MDGs that could play a crucial role in influencing the immune microenvironment. In summary, we experimented with various thresholds and selected one suitable for our study. Although it may impact sensitivity, we believe it is acceptable considering the influence of gene expression regulation by DNA methylation. Subsequently, consensus clustering was conducted to identify distinct molecular clusters of colon cancers based on the methylation data. The patients of colon cancer were then divided into four clusters, and the immune microenvironment of each cluster was further analyzed. Notably, patients in Cluster 1, characterized by stronger antitumor immunoactivity, were predicted to have a better response to immunotherapy based on the Tide score. Finally, we identified the specific methylation markers of Cluster1 (PCDH20, APCDD1, COCH), and ROC curves confirmed their excellent performance in discriminating the clusters.

There were several factors could affect the effectiveness of immunotherapy in colon cancers. The composition and quantity of infiltrating immune cells in the TIME played crucial roles in the process of tumor eradication (34). The infiltration of CD8+ T cells or CTLs had a significant positive association with antitumor immune activity (35). On the other hand, Tregs could induce the apoptosis of cytotoxic T cells, leading to the immunosuppression (36, 37). In the present study, we statistically analyzed the infiltration abundance of immune cells from clusters. It was observed that Cluster 1 had a higher abundance of CD8+ T cells and CTL, while the infiltration of Tregs was found to be the lowest among the clusters (*P*<0.05). Other factors that affect immunotherapy include the expression of ICIs, TMB, etc. (38, 39) Compared with Cluster 2 and 3, the expression levels of ICIs were significantly higher in Cluster 1. Additionally, patients in Cluster 1 had significantly higher TMB than those in remaining clusters (*P*<0.05). The Tide scores indicated that Cluster 1 was most likely to benefit from immunotherapy. Notably, the distribution frequency of BRAF mutation (70.9%) and MSI-H (52.3%) in Cluster 1 were significantly higher than that in other clusters. There was a high overlap of 77.2% between these two groups of patients. BRAF is a serine/threonine protein kinase located downstream of RAS/RAF/MAPK pathway (40). The BRAF mutation, (primarily caused by a missense mutation at V600E) was a significant mutation in colon cancers. The relationship between BRAF mutation and MSI-H has been extensively discussed. It has been confirmed that patients with BRAF mutations have a higher rate of MSI-H. This may be due to the tumors with BRAF V600E mutation were associated with a high-level CpG island methylator phenotype (CIMP) and MLH1 promoter methylation (41, 42). However, the impact of BRAF mutation on immunotherapy response in dMMR patients has always been controversial. A recent retrospective study concluded that there were no significant differences in neoantigen tumor burden (NTB), immune score, or T cell infiltration between BRAF wild-type and mutant of colon cancer patients with MSI-H (43). This suggested that both are likely to benefit from immune checkpoint inhibitors. In conclusion, Cluster 1, which has a higher frequency of BRAF mutation and MSI-H, is more suitable for immunotherapy based on the TIME analysis.

We successfully identified specific methylation markers (PCDH20, APCDD1, COCH) of immune-beneficial cluster using the QDMR software. As a tumor suppressor gene, protocadherin 20 (PCDH20) is a member of the cadherin superfamily (44). The previous studies have shown that the expression of PCDH20 was frequently decreased or silenced in multiple cancers, primarily attributed to the methylation of the promoter region. The expression of PCDH20 was restored after the addition of DNMT inhibitors to the corresponding tumor cell lines (45, 46). In addition, it has been observed that inhibition of PCDH20 expression frequently promoted migration and invasion of tumors (47). Notably, PCDH20 plays a crucial role in maintaining the balance and structural integrity of the intestinal epithelium. A decrease in the expression level of PCDH20 can disrupt the integrity of the intestinal mucosa, which can contribute to the development of colitis and Crohn’s disease (48). APCDD1 (adenomatosis polyposis down-regulated 1), a negative regulator of Wnt/β-catenin pathway, its expression was regulated by promotor methylation (49). It has been demonstrated that the methylation of WNT target genes (including APCDD1) could be serve as reliable biomarkers for predicting recurrence in colon cancers (50). As a DNA methylation marker, COCH has shown effectiveness in identifying occult lymph node metastases in non-small cell lung cancer (51). However, the effect of promoter methylation on the expression of COCH has not been extensively studied. In contrast to previous research, this study was the first to discuss the differences in methylation levels of markers (PCDH20, APCDD1, COCH) among different clusters. The methylation levels of the three specific methylation markers in Cluster 1 were found to be significantly distinct from those in the other clusters. In this study, we utilized colon cancer samples (immunohistochemistry) to validate the conclusion. However, we did not observe any significant difference in the expression of COCH between response group and non-response group. This might be attributed to the markers being associated with clustering, while the potential mechanisms related to the TIME remain unconfirmed.

DNA methylation biomarkers exhibited a better sensitivity compared to mutation-based cancer detection (52–54). Currently, DNA methylation markers are predominantly utilized as diagnostic and prognostic markers. The innovation of this study lies in exploring biomarkers of immunotherapy in colon cancers from the perspective of DNA methylation. Ultimately, specific methylation markers (PCDH20, APCDD1, and COCH) were identified as effective markers for identifying cluster that would benefit from immunotherapy in colon cancers. Our study still had some limitations. Firstly, the sample size used in the study was mainly derived from the database, we performed immunohistochemical validation of small samples to verify the research findings. However, for further validation, large sample sequencing data will be required in the future. Secondly, the potential mechanisms linking molecular markers and immune status has not been fully elucidated. Lastly, we will concentrate on assessing the markers’ feasibility in clinical practice and making further enhancements and optimizations.

## 5 Conclusion

In conclusion, this study successfully identified a specific cluster that benefited from immunotherapy through 282 MDGs of colon cancers. Furthermore, we found beneficial-cluster of specific methylation markers (PCDH20, APCDD1, COCH) that could be used in conjunction with microsatellite status to expand the pool of colon cancer patients eligible for immunotherapy.

## Data availability statement

The original contributions presented in the study are included in the article/**Supplementary Materials**, further inquiries can be directed to the corresponding authors.

## Ethics statement

The studies involving humans were approved by Harbin Medical University Cancer Hospital ethics committee. The studies were conducted in accordance with the local legislation and institutional requirements. The human samples used in this study were acquired from primarily isolated as part of your previous study for which ethical approval was obtained. Written informed consent for participation was not required from the participants or the participants’ legal guardians/next of kin in accordance with the national legislation and institutional requirements.

## Author contributions

BX: Conceptualization, Data curation, Formal Analysis, Investigation, Methodology, Resources, Software, Validation, Visualization, Writing – original draft, Writing – review & editing. JL: Data curation, Methodology, Software, Writing – review & editing. XP: Data curation, Writing – review & editing. YG: Methodology, Software, Writing – review & editing. JZ: Formal Analysis, Writing – review & editing. YZ: Conceptualization, Project administration, Resources, Supervision, Writing – review & editing. HL: Funding acquisition, Project administration, Supervision, Writing – review & editing.
