# Supplementary material for: Identification of colon cancer subtypes based on multi-omics data—construction of methylation markers for immunotherapy
Source: Front Oncol. 2024 Jan 22;14:1335670. doi: 10.3389/fonc.2024.1335670 (PMC10848914; doi:10.3389/fonc.2024.1335670)
Supplement: Supplementary file 1 [file DataSheet_1.zip › supplementary figure legend and table titles.docx]

**Figure S1** Consensus matrix for DNA methylation classification and analysis of tumor immune in validation cohort. **(A)** Consensus cumulative distribution function (CDF) of different clusters. **(B)** Delta area curve of consensus clustering. **(C)** Consensus clustering matrix for colon cancer at k = 4. **(D)** The survival curves in four DNA methylation clusters. **(E, F)** Comparisons of stromal score, immune score, ESTIMATE score and tumor purity during four clusters. **(G)** The expression level of immune checkpoints (PD1, PDL1, PDL2, CTLA4, LAG3) in the four clusters of colon cancer. ‘ns’ means *p* > 0.05, * means *p*< 0.05, ** means *p*< 0.01, *** means *p* < 0.001.

**Figure S2** Somatic variations features during clusters and predicting the response to immune therapy based on tumor immune dysfunction and exclusion (TIDE) in validation cohort. **(A)** The abundance of immune cells in the four clusters of colon cancer patients evaluated by CIBERSORT algorithm. **(B)** Comparisons of mutation status of APC, KRAS, BRAF, TP53 and PIK3CA during different clusters of colon cancer patients. **(C)** Comparisons of tumor mutation burdens (TMB) level of the four clusters. **(D)** The distribution frequency of microsatellite status during four clusters. **(E)** Comparisons of cytotoxic T lymphocytes (CTL) level of the four clusters. **(F)** Comparisons of Tide score for predicting the likelihood of response to immune therapy of different clusters. ‘ns’ means *p* > 0.05, * means *p*< 0.05, ** means *p*< 0.01, *** means *p* < 0.001.

**Figure S3** The correlation coefficients between DNA methylation and gene expression by Pearson correlation analysis. **(A)** PCDH20. **(B)** APCDD1. **(C)** COCH.

**Figure S4** The survival analysis of specific markers (APCDD1, COCH, PCDH20) by the Kaplan–Meier method. **(A)** The survival curves of APCDD1 based on gene expression. **(B)** The survival curves of COCH based on gene expression. **(C)** The survival curves of PCDH20 based on gene expression. **(D)** The survival curves of APCDD1 based on DNA methylation. **(E)** The survival curves of COCH based on DNA methylation. **(F)** The survival curves of PCDH20 based on DNA methylation.

**Figure S5** The distribution level of APCDD1 in different stages of colon cancers. **(A)** Methylation levels of APCDD1 were assessed in different stages of colon cancers. (B) Expression levels of APCDD1 mRNA were assessed in different stages of colon cancers. ‘ns’ means *p* > 0.05, * means *p*< 0.05, ** means *p*< 0.01, *** means *p* < 0.001.

**Figure S6** The association between markers (COCH, PCDH20) and immune status by Pearson correlation analysis. **(A)** The methylation level of COCH and immune scores. **(B)** The methylation level of COCH and CD8 T+ cells. **(C)** The methylation level of COCH and PD-1 expressions. **(D)** The methylation level of COCH and PD-L1 expressions. **(E)** The methylation level of APCDD1 and immune scores. **(F)** The methylation level of APCDD1 and CD8 T+ cells. **(G)** The methylation level of APCDD1 and PD-1 expressions. **(H)** The methylation level of APCDD1 and PD-L1 expressions.

**Table S1** 2217 of differentially expressed genes with aberrantly methylation.

**Table S2** 282 DNA methylation driver genes in colon cancer samples.

**Table S3** Clinical characteristics of training and validation cohort.

**Table S4** Baseline clinical and pathological characteristics of patients with different cluster in training cohort.

**Table S5** Baseline clinical and pathological characteristics of patients with different cluster in validation cohort.

**Table S6** 56 specific methylation genes of four clusters.

**Table S7** Characteristics of clinical validation samples.

**Table S8** 2058 of differentially methylation genes based on APCDD1 groups
