# Supplementary material for: Identification of colon cancer subtypes based on multi-omics data—construction of methylation markers for immunotherapy
Source: Front Oncol. 2024 Jan 22;14:1335670. doi: 10.3389/fonc.2024.1335670 (PMC10848914; doi:10.3389/fonc.2024.1335670)
Supplement: Supplementary file 1 [file DataSheet_1.zip › Table S2 and 3.docx]

**Table S2. 282 DNA methylation driver genes in colon cancer samples**

| **Gene name** | **The correlation between DNA-methylation and gene expression data** |
| --- | --- |
| ABCC2 | -0.495 |
| ABHD6 | -0.387 |
| ACVRL1 | -0.534 |
| ADHFE1 | -0.352 |
| AIFM3 | -0.587 |
| AIM2 | -0.313 |
| ANO5 | -0.383 |
| ANO10 | -0.343 |
| AOX1 | -0.357 |
| APCDD1 | -0.309 |
| APCDD1L | -0.398 |
| AQP1 | -0.366 |
| AQP5 | -0.549 |
| ARMC2 | -0.704 |
| ATP8B1 | -0.410 |
| ATP11A | -0.341 |
| AZGP1 | -0.408 |
| B3GALT4 | -0.361 |
| B3GNT7 | -0.322 |
| BATF | -0.658 |
| BCL2L15 | -0.337 |
| BST2 | -0.698 |
| C2 | -0.416 |
| C2CD4A | -0.306 |
| C4orf19  C5orf38  C10orf99  CAPN9  CARD14  CCDC152  CCL25  CCNA1  CD3E  CD3G  CD46  CDC42EP5  CDH16  CDO1  CEACAM6 | -0.375  -0.625  -0.686  -0.480  -0.417  -0.345  -0.453  -0.321  -0.339  -0.365  -0.307  -0.396  -0.349  -0.410  -0.537 |
|  |  |

**Table S2. 282 DNA methylation driver genes (continued)**

| **Gene name** | **The correlation between DNA-methylation and gene expression data** |
| --- | --- |
| CEL | -0.540 |
| CES3 | -0.575 |
| CHL1  CHN2  CHST4  CKB  CLDN10  CLIC6  CLIP3  CLIP4  CNRIP1  COCH | -0.310  -0.430  -0.361  -0.373  -0.406  -0.583  -0.567  -0.428  -0.477  -0.329 |
| CPNE8  CRYAB  DBNDD1  DMRTA1  DOK2 | -0.372  -0.391  -0.324  -0.385  -0.440 |
| DOLPP1  DPP6  DPP10  EBPL | -0.494  -0.311  -0.525  -0.316 |
| ECT2  EFS  EIF5A2  ELF5  EML1  ENGASE  EPHX3  EPHX4  EREG  EVPL  F2 | -0.357  -0.341  -0.322  -0.687  -0.326  -0.463  -0.342  -0.471  -0.513  -0.389  -0.346 |
| FADS2  FAM43B  FBLIM1  FCGBP  FCHO1 | -0.362  -0.353  -0.450  -0.706  -0.510 |
|  |  |
|  |  |
|  |  |

**Table S2. 282 DNA methylation driver genes (continued)**

| **Gene name** | **The correlation between DNA-methylation and gene expression data** |
| --- | --- |
| FERMT1  FGF2  FGF10  FLI1  FLRT2 | -0.324  -0.543  -0.315  -0.321  -0.331 |
| FOXD2  FOXG1  FOXH1  FOXRED2  FZD10  GALNT6  GATA4  GJB3  GJB5  GJC2  GOLGA8A  GPRIN2  GPSM2  GREB1L  GSTM5  GYPC  HABP2  HCRT  HMGA1  HS3ST3A1 | -0.522  -0.436  -0.660  -0.408  -0.443  -0.458  -0.344  -0.334  -0.742  -0.538  -0.345  -0.334  -0.440  -0.399  -0.506  -0.647  -0.405  -0.588  -0.581  -0.341 |
| HTR4  HYDIN  ICA1  IFITM1  IL1RN  IL10  IL20RA  INPP5D  INSL4  IQCA1  IRF4  IRX2  ISX  JAM3 | -0.377  -0.332  -0.358  -0.500  -0.354  -0.360  -0.355  -0.714  -0.376  -0.467  -0.307  -0.611  -0.743  -0.379 |

**Table S2. 282 DNA methylation driver genes (continued)**

| **Gene name** | **The correlation between DNA-methylation and gene expression data** |
| --- | --- |
| KANK2  KCNA3  KIF25  KLK6  KRT7  KRT20  KRT23  KRT40  LGALS4  LIMA1  LRFN5  LRRC31  LY6G6E  MAB21L2  MAL  MALL  MAT1A  MEL1 | -0.330  -0.412  -0.647  -0.310  -0.313  -0.338  -0.584  -0.332  -0.416  -0.345  -0.323  -0.333  -0.408  -0.638  -0.345  -0.440  -0.301  -0.369 |
| MET | -0.361 |
| MEX3A | -0.472 |
| MFAP4 | -0.392 |
| MIOX | -0.330 |
| MME | -0.317 |
| MSLN | -0.357 |
| MUC12 | -0.465 |
| MUC17 | -0.500 |
| MYBPHL | -0.375 |
| MYEOV | -0.419 |
| MYO1A | -0.348 |
| NANOS3 | -0.600 |
| NEFM | -0.354 |
| NEU4 | -0.320 |
| NKAPL | -0.433 |
| NOD2 | -0.413 |
| NPC1L1 | -0.794 |
| NPEPL1 | -0.390 |
| NPSR1 | -0.550 |
| NPTX2 | -0.437 |
| NPY | -0.311 |

**Table S2. 282 DNA methylation driver genes (continued)**

| **Gene name** | **The correlation between DNA-methylation and gene expression data** |
| --- | --- |
| NR3C2  NRARP  OAS2  OSBPL3  P2RY2  PALM  PAQR5  PCDH17  PCDH20  PCDHB8  PCSK9  PDE10A  PDRG1  PDX1  PDZK1  PER3  PGM1  PIGR | -0.341  -0.319  -0.301  -0.406  -0.325  -0.304  -0.423  -0.407  -0.335  -0.347  -0.336  -0.341  -0.322  -0.306  -0.451  -0.486  -0.478  -0.706 |
| PIPOX | -0.409 |
| PITX1 | -0.314 |
| PKP1 | -0.349 |
| PLAGL2 | -0.445 |
| PLEKHG4 | -0.677 |
| PLEKHG6 | -0.463 |
| PLEKHN1 | -0.314 |
| PMEPA1 | -0.536 |
| PPP1R16B | -0.313 |
| PROM2 | -0.500 |
| PRSS36 | -0.340 |
| PTF1A | -0.352 |
| PTK7 | -0.419 |
| PTPRF | -0.355 |
| RARRES2 | -0.443 |
| RBP1 | -0.457 |
| RCN1 | -0.372 |
| RELL2 | -0.342 |
| REP15 | -0.395 |
| RGR | -0.365 |
| RIBC2 | -0.365 |

**Table S2. 282 DNA methylation driver genes (continued)**

| **Gene name** | **The correlation between DNA-methylation and gene expression data** |
| --- | --- |
| RIC3  RNF43  RPS6KL1  RSPO2  S100P  SEMA4G  SERPINA1  SERPIND1  SGK2  SH3TC2  SLC5A6  SLC6A6  SLC9A1  SLC13A3  SLC23A1  SLCO4A1  SLIT2  SLIT3 | -0.340  -0.503  -0.434  -0.311  -0.333  -0.365  -0.515  -0.301  -0.459  -0.396  -0.699  -0.574  -0.319  -0.443  -0.301  -0.309  -0.433  -0.360 |
| SMOC1 | -0.363 |
| SNCA | -0.342 |
| SOX1 | -0.400 |
| SOX14 | -0.426 |
| SOX15 | -0.382 |
| SOX17 | -0.443 |
| SPDYC | -0.352 |
| SPTBN5 | -0.382 |
| ST6GALNAC5 | -0.423 |
| STAC | -0.416 |
| STC2 | -0.556 |
| STK31 | -0.600 |
| STK32B | -0.327 |
| STRA6 | -0.473 |
| STRADB | -0.394 |
| SULT1C2 | -0.516 |
| SUSD5 | -0.330 |
| SYTL1 | -0.526 |
| TBX18 | -0.699 |
| TBX20 | -0.419 |
| TCN1 | -0.316 |

**Table S2. 282 DNA methylation driver genes (continued)**

| **Gene name** | **The correlation between DNA-methylation and gene expression data** |
| --- | --- |
| TFAP2C  TGFB1I1  TINAG  TMEM25  TMEM37  TMEM63A  TMEM155  TMEM220  TMTC1  TOMM34  TPM1  TRIM29  TRPC6  TUBAL3  TUSC3  TWIST1  UNC5C  UNC5CL | -0.380  -0.320  -0.424  -0.578  -0.326  -0.402  -0.317  -0.560  -0.376  -0.338  -0.420  -0.550  -0.389  -0.506  -0.499  -0.370  -0.364  -0.552 |
| VDR | -0.449 |
| VENTX | -0.313 |
| VIPR2 | -0.373 |
| VSIG2 | -0.625 |
| WDR17 | -0.448 |
| XCR1 | -0.336 |
| ZBTB7B | -0.307 |
| ZFP3 | -0.533 |
| ZIK1 | -0.580 |
| ZNF135 | -0.644 |
| ZNF229 | -0.438 |
| ZNF280A | -0.335 |
| ZNF280B | -0.527 |
| ZNF304 | -0.772 |
| ZNF385D | -0.321 |
| ZNF415 | -0.731 |
| ZNF418 | -0.405 |
| ZNF454 | -0.378 |
| ZNF471 | -0.656 |
| ZNF492 | -0.539 |
| ZNF528 | -0.673 |

**Table S2. 282 DNA methylation driver genes (continued)**

| **Gene name** | **The correlation between DNA-methylation and gene expression data** |
| --- | --- |
| ZNF556  ZNF568  ZNF582  ZNF626  ZNF667  ZNF671  ZNF677  ZNF772  ZNF829  ZSCAN18 | -0.463  -0.494  -0.564  -0.390  -0.467  -0.460  -0.560  -0.510  -0.580  -0.704 |

**Table S3. Clinical characteristics of training and validation cohort.**

| **Clinical characteristics** | **Training cohort (n=197)** | **Validation cohort (n=85)** | ***P* value** |
| --- | --- | --- | --- |
| Gender |  |  | *P*=0.46 |
| Male | 109 (55.4%) | 43 (49.5%) |  |
| Female | 88 (44.6%) | 42 (50.6%) |  |
| Age |  |  | *P*=0.83 |
| ≤65 | 93 (47.2%) | 39 (45.9%) |  |
| ＞65 | 104 (52.8%) | 46 (54.1%) |  |
| T stage |  |  | *P*=0.70 |
| T1 | 5 (2.6%) | 3 (3.5%) |  |
| T2 | 33 (16.7%) | 10 (11.7%) |  |
| T3 | 132 (67.0%) | 61 (71.7%) |  |
| T4 | 27 (13.7%) | 11 (13.1%) |  |
| N stage |  |  | *P*=0.76 |
| N0 | 113 (57.3%) | 52 (61.2%) |  |
| N1 | 52 (26.5%) | 19 (22.3%) |  |
| N2 | 32 (16.2) | 14 (16.5%) |  |
| M stage |  |  | *P*=0.66 |
| M0 | 167 (84.7%) | 72 (84.7%) |  |
| M1 | 28 (14.3%) | 11 (13.0%) |  |
| Mx | 2 (1.0%) | 2 (2.3%) |  |
| TNM stage |  |  | *P*=0.77 |
| I | 33 (16.7%) | 11 (12.9%) |  |
| II | 76 (38.5%) | 37 (43.6%) |  |
| III | 58 (29.5%) | 24 (28.2%) |  |
| IV | 28 (14.3%) | 11 (12.9%) |  |
| Unknown | 2 (1.0%) | 2 (2.3%) |  |
| **Microsatellite status** |  |  | *P*=0.36 |
| **MSS** | 141 (71.5%) | 67 (78.8%) |  |
| MSI-H | 42 (21.4%) | 12 (14.2%) |  |
| Unknown | 14 (7.1%) | 6 (7.0%) |  |
| Cluster |  |  | *P*=0.13 |
| 1 | 44 (22.4%) | 13 (15.4%) |  |
| 2 | 70 (35.5%) | 25 (29.4%) |  |
| 3 | 52 (26.4%) | 25 (29.4%) |  |
| 4 | 31 (15.7%) | 22 (25.8%) |  |

Abbreviations: tumor node metastasis, TNM; microsatellite stability, MSS;

microsatellite instability – high, MSI-H.
