# Supplementary material for: Identification of colon cancer subtypes based on multi-omics data—construction of methylation markers for immunotherapy
Source: Front Oncol. 2024 Jan 22;14:1335670. doi: 10.3389/fonc.2024.1335670 (PMC10848914; doi:10.3389/fonc.2024.1335670)
Supplement: Supplementary file 1 [file DataSheet_1.zip › Table S4.docx]

**Table S4. Baseline clinical and pathological characteristics of patients with different cluster in training cohort.**

| **Characteristics** | **Cluster 1** | **Cluster 2** | **Cluster 3** | **Cluster 4** | ***P* value** |
| --- | --- | --- | --- | --- | --- |
|  | **(N = 44)** | **(N = 70)** | **(N = 52)** | **(N=31)** |  |
| Gender |  |  |  |  | 0.85 |
| Male | 25 (56.9%) | 37 (52.8%) | 31 (59.6%) | 16 (51.6%) |  |
| Female | 19 (43.1%) | 33 (47.2%) | 21 (40.4%) | 15 (48.4%) |  |
| Age |  |  |  |  | ＜0.001 |
| ≤65 | 14 (31.8%) | 37 (52.8%) | 20 (38.5%) | 22 (70.9%) |  |
| ＞65 | 30 (68.2%) | 33 (47.2%) | 32 (61.5%) | 9 (29.1%) |  |
| T stage |  |  |  |  | 0.44 |
| T1 | 1 (2.2%) | 3 (4.2%) | 1 (1.9%) | 0 (0.0%) |  |
| T2 | 11 (25.0%) | 12 (17.1%) | 9 (17.3%) | 1 (3.2%) |  |
| T3 | 28 (63.6%) | 45 (64.2%) | 34 (65.3%) | 25 (80.6%) |  |
| T4 | 4 (9.2%) | 10 (14.5%) | 8 (15.5%) | 5 (16.2%) |  |
| N stage |  |  |  |  | 0.32 |
| N0 | 27 (61.3%) | 35 (50.0%) | 33 (63.4%) | 18 (58.0%) |  |
| N1 | 11 (25.0%) | 25 (35.5%) | 8 (15.5%) | 8 (25.8%) |  |
| N2 | 6 (13.7%) | 10 (14.5%) | 11 (21.1%) | 5 (16.2%) |  |
| M stage |  |  |  |  |  |
| M0 | 39 (88.6%) | 55 (78.6%) | 47 (90.3%) | 26 (83.8%) | 0.43 |
| M1 | 5 (11.4%) | 14 (20.0%) | 5 (9.7%) | 4 (13.0%) |  |
| Mx | 0 (0.0%) | 1 (1.4%) | 0 (0.0%) | 1 (3.2%) |  |
| TNM stage |  |  |  |  | 0.25 |
| I | 11 (25.0%) | 11 (15.8%) | 10 (19.2%) | 1 (3.2%) |  |
| II | 15 (34.1%) | 22 (31.4%) | 23 (44.2%) | 16 (51.6%) |  |
| III | 13 (29.5%) | 22 (31.4%) | 14 (26.9%) | 9 (29.1%) |  |
| IV | 5 (11.4%) | 14 (20.0%) | 5 (9.7%) | 4 (12.9%) |  |
| Unknown | 0 (0.0%) | 1 (1.4%) | 0 (0.0%) | 1 (3.2%) |  |
| Microsatellite status |  |  |  |  | ＜0.001 |
| MSS | 19 (43.1%) | 58 (82.8%) | 46 (88.5%) | 18 (58.1%) |  |
| MSI-H | 22 (50.0%) | 4 (5.7%) | 4 (7.7%) | 12 (38.7%) |  |
| Unknown | 3 (6.9%) | 8 (11.5%) | 2 (3.8%) | 1 (3.2%) |  |

Abbreviations: TNM, tumor node metastasis; MSS, microsatellite stability;

MSI-H, Microsatellitein stability – High.
