# Supplementary material for: Identification of colon cancer subtypes based on multi-omics data—construction of methylation markers for immunotherapy
Source: Front Oncol. 2024 Jan 22;14:1335670. doi: 10.3389/fonc.2024.1335670 (PMC10848914; doi:10.3389/fonc.2024.1335670)
Supplement: Supplementary file 1 [file DataSheet_1.zip › Table S5.docx]

**Table S5. Baseline clinical and pathological characteristics of patients with different cluster in validation cohort.**

| **Characteristics** | **Cluster 1** | **Cluster 2** | **Cluster 3** | **Cluster 4** | ***P* value** |
| --- | --- | --- | --- | --- | --- |
|  | **(N = 13)** | **(N = 25)** | **(N = 25)** | **(N=22)** |  |
| Gender |  |  |  |  | 0.45 |
| Male | 4 (30.7%) | 14 (56.0%) | 14 (56.0%) | 11 (50.0%) |  |
| Female | 9 (69.3%) | 11 (44.0%) | 11 (44.0%) | 11 (50.0%) |  |
| Age |  |  |  |  | ＜0.05 |
| ≤65 | 3 (23.1%) | 13 (52.0%) | 10 (40.0%) | 13 (59.1%) |  |
| ＞65 | 10 (76.9%) | 12 (48.0%) | 15 (60.0%) | 9 (40.9%) |  |
| T stage |  |  |  |  | 0.94 |
| T1 | 1 (7.7%) | 1 (4.0%) | 0 (1.9%) | 1 (4.5%) |  |
| T2 | 1 (7.7%) | 3 (12.0%) | 4 (16.0%) | 2 (9.1%) |  |
| T3 | 9 (69.2%) | 19 (76.0%) | 18 (72.0%) | 15 (68.2%) |  |
| T4 | 2 (15.4%) | 2 (8.0%) | 3 (12.0%) | 4 (18.2%) |  |
| N stage |  |  |  |  | 0.53 |
| N0 | 11 (84.6%) | 16 (64.0%) | 13 (52.0%) | 12 (54.5%) |  |
| N1 | 1 (7.7%) | 6 (24.0%) | 6 (24.0%) | 6 (27.3%) |  |
| N2 | 1 (7.7%) | 3 (12.0%) | 6 (24.0%) | 4 (18.2%) |  |
| M stage |  |  |  |  | 0.30 |
| M0 | 13 (100.0%) | 19 (76.0%) | 20 (80.0%) | 20 (91.0%) |  |
| M1 | 0 (0.0%) | 5 (20.0%) | 5 (20.0%) | 1 (4.5%) |  |
| Mx | 0 (0.0%) | 1 (4.0%) | 0 (0.0%) | 1 (4.5%) |  |
| TNM stage |  |  |  |  |  |
| I | 2 (15.3%) | 3 (12.0%) | 3 (12.0%) | 3 (13.6%) | 0.34 |
| II | 9 (69.4%) | 11 (44.0%) | 9 (36.0%) | 8 (36.3%) |  |
| III | 2 (15.3%) | 5 (20.0%) | 8 (32.0%) | 9 (41.1%) |  |
| IV | 0 (0.0%) | 5 (20.0%) | 5 (20.0%) | 1 (4.5%) |  |
| Unknown | 0 (0.0%) | 1 (4.0%) | 0 (0.0%) | 1 (4.5%) |  |
| Microsatellite status |  |  |  |  | ＜0.001 |
| MSS | 3 (23.1%) | 22 (88.0%) | 23 (92.0%) | 19 (86.4%) |  |
| MSI-H | 8 (61.6%) | 0 (5.7%) | 1 (4.0%) | 3 (13.6%) |  |
| Unknown | 2 (15.3%) | 3 (12.0%) | 1 (4.0%) | 0 (3.2%) |  |

Abbreviations: TNM, tumor node metastasis; MSS, microsatellite stability;

MSI-H, Microsatellitein stability – High.
