# Supplementary figures and images for: Identification of colon cancer subtypes based on multi-omics data—construction of methylation markers for immunotherapy
Source: Front Oncol. 2024 Jan 22;14:1335670. doi: 10.3389/fonc.2024.1335670 (PMC10848914; doi:10.3389/fonc.2024.1335670)

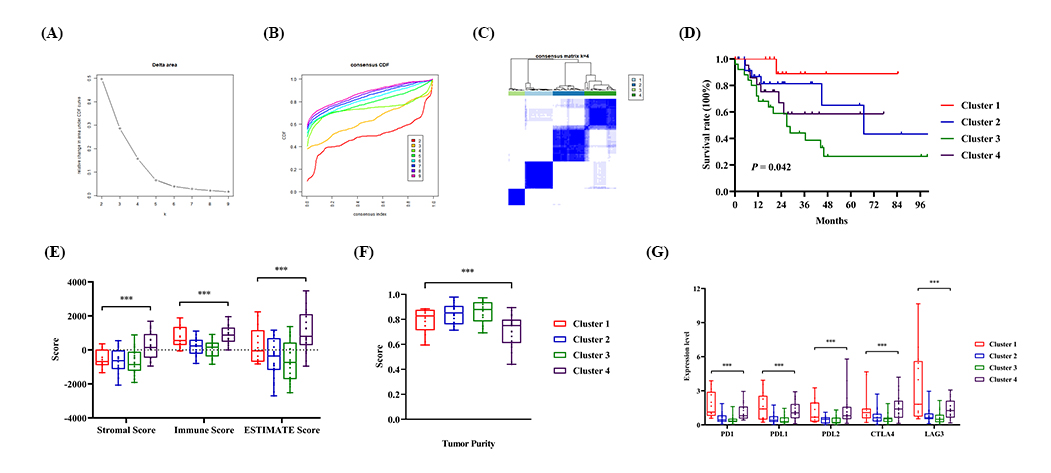

Supplement: Supplementary file 1 [file DataSheet_1.zip › Figure S1.jpg]

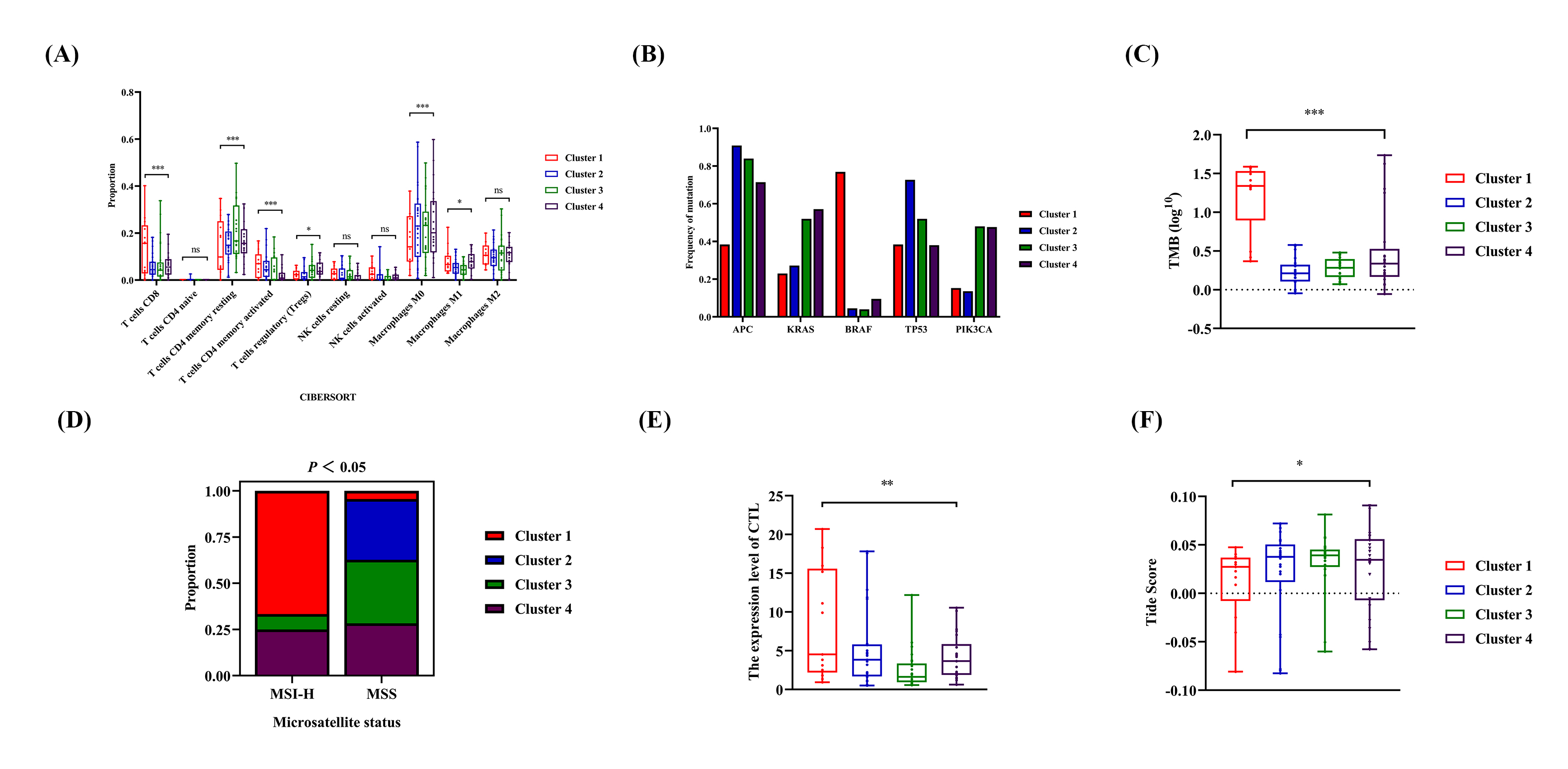

Supplement: Supplementary file 1 [file DataSheet_1.zip › Figure S2.jpg]

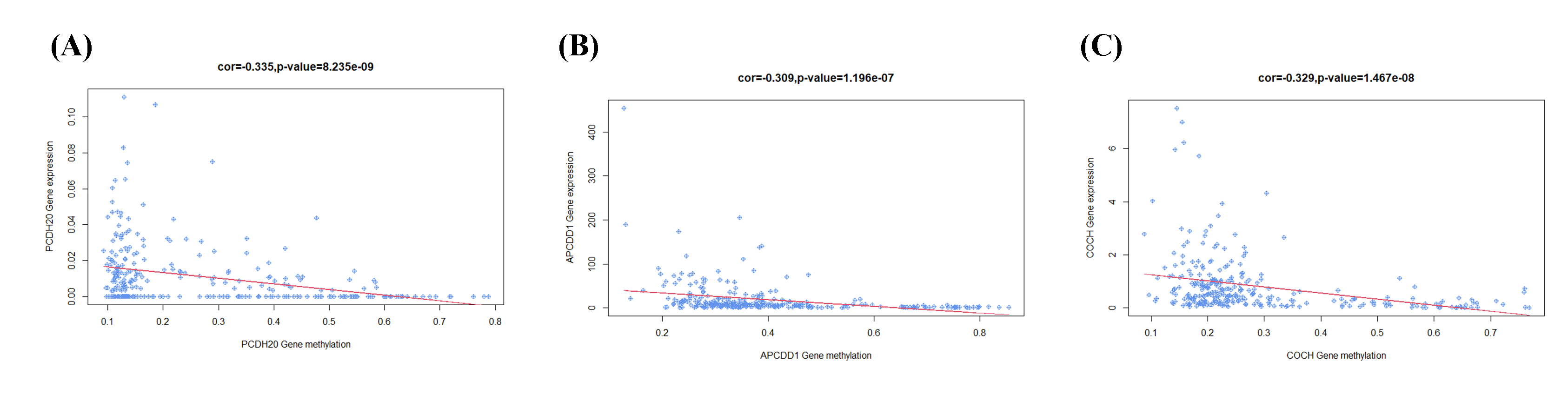

Supplement: Supplementary file 1 [file DataSheet_1.zip › Figure S3.jpg]

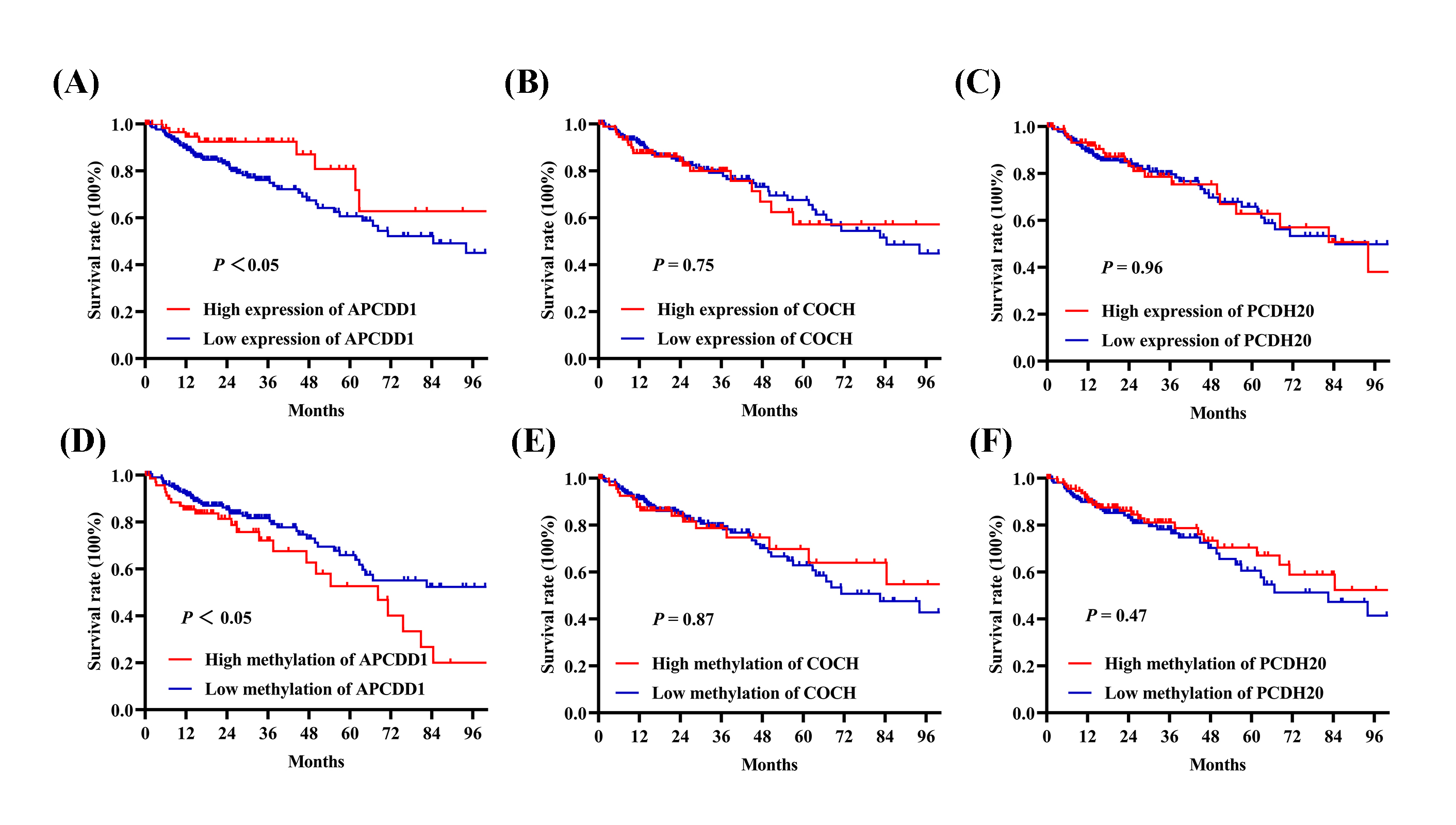

Supplement: Supplementary file 1 [file DataSheet_1.zip › Figure S4.jpg]

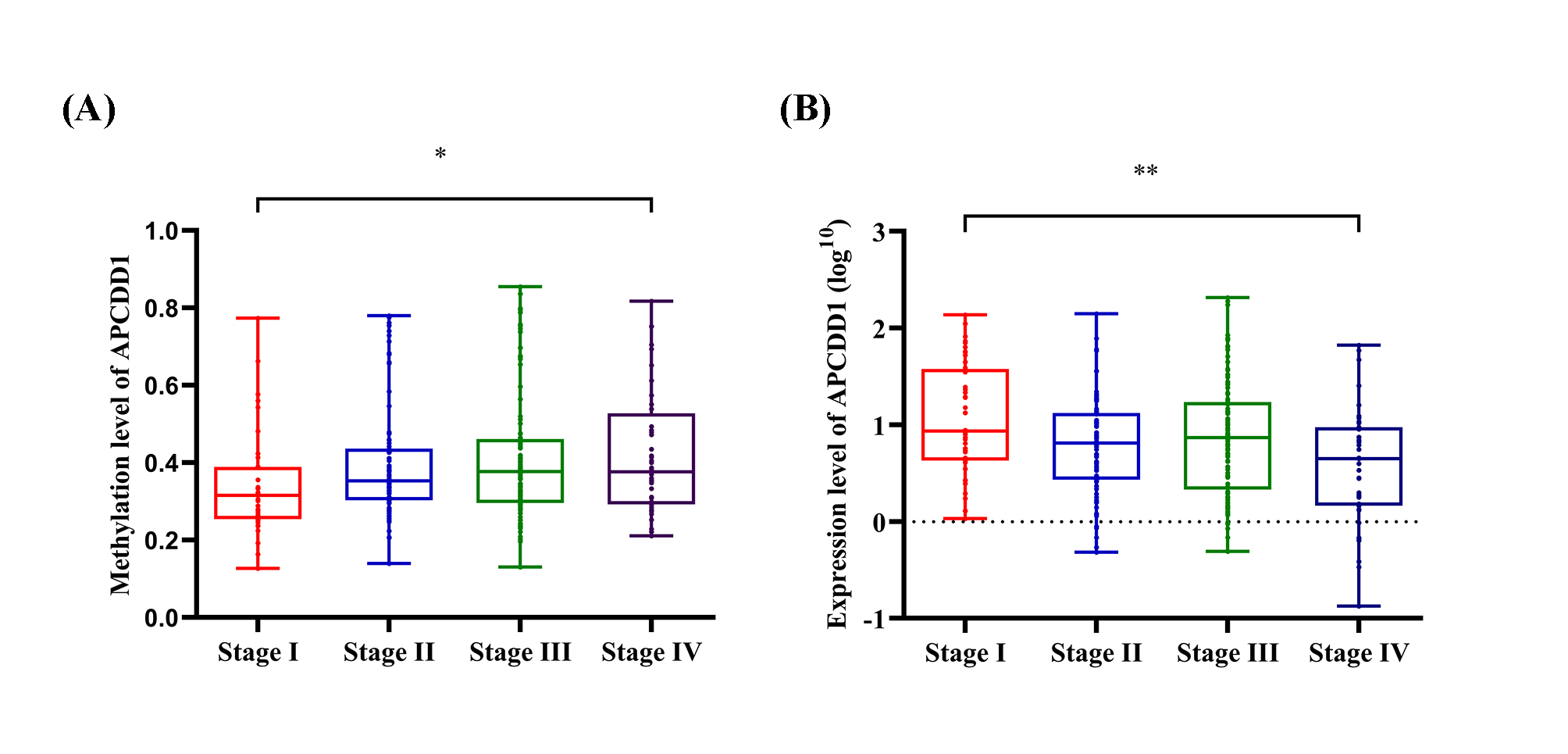

Supplement: Supplementary file 1 [file DataSheet_1.zip › Figure S5.jpg]

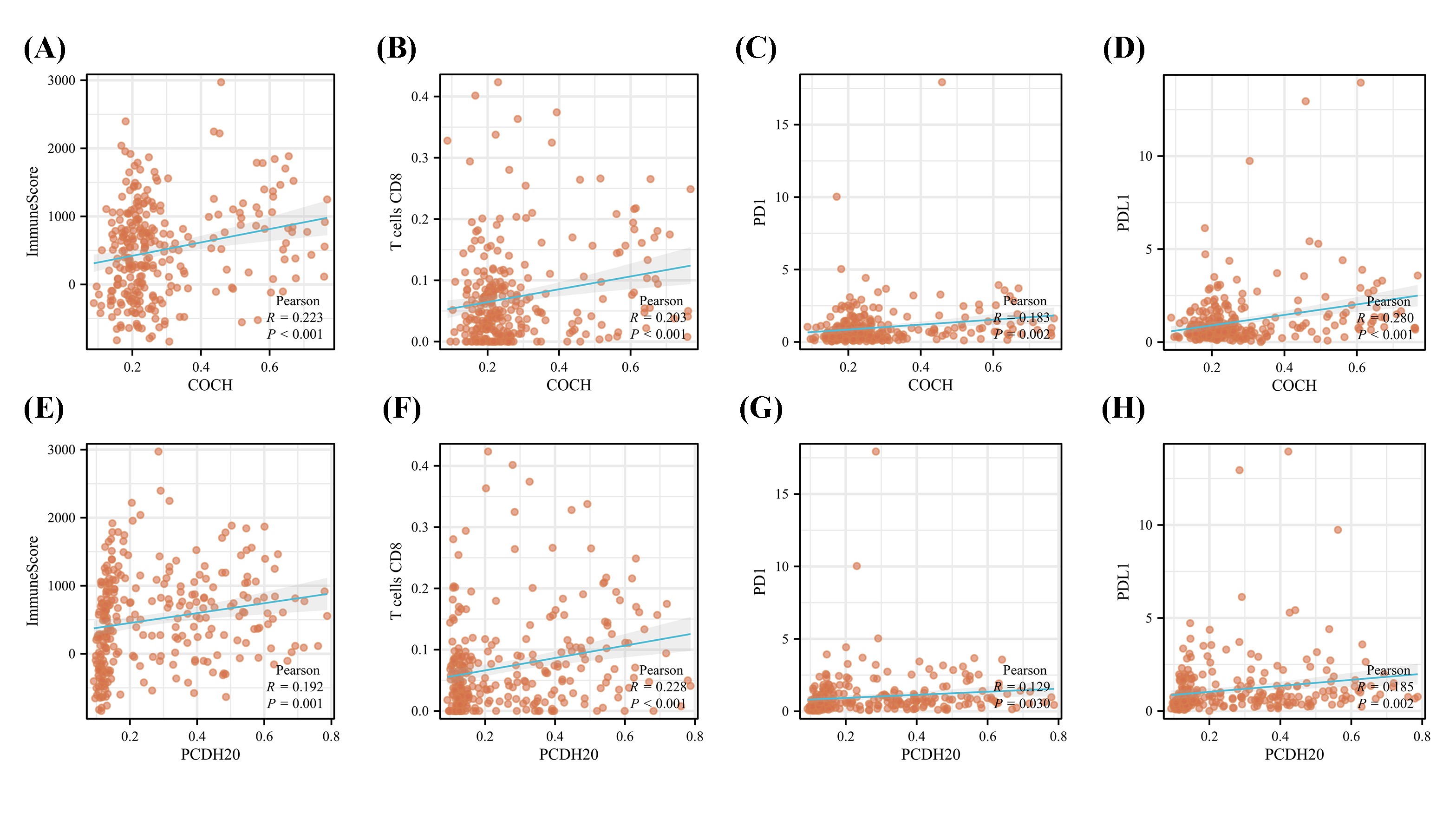

Supplement: Supplementary file 1 [file DataSheet_1.zip › Figure S6.jpg]
